# Comparative analyses of salivary exosomal miRNAs for patients with or without lung cancer

**DOI:** 10.3389/fgene.2023.1249678

**Published:** 2023-11-03

**Authors:** Mengfeng Liu, Xiran Yu, Jianlong Bu, Qifan Xiao, Sitong Ma, Naozhong Chen, Changfa Qu

**Affiliations:** Department of Thoracic Surgery, Harbin Medical University Cancer Hospital, Harbin, Heilongjiang, China

**Keywords:** lung cancer, saliva, exosome, microRNA, miRNA sequencing, biomarker

## Abstract

**Introduction:** Lung cancer is the most frequent cause of cancer-related deaths worldwide. Exosomes are involved in different types of cancer, including lung cancer.

**Methods:** We collected saliva from patients with (LC) or without (NC) lung cancer and successfully isolated salivary exosomes by ultracentrifugation. MiRNA sequencing was implemented for the exosome samples from NC and LC groups, dgeR was used to determine differentially expressed miRNAs (DE miRNAs), and quantitative real-time polymerase chain reaction (qPCR) was used to verify three differentially expressed microRNAs (miRNAs).

**Results:** A total of 372 miRNAs were identified based on the sequencing results. Subsequently, 15 DE miRNAs were identified in LC vs. NC, including eight upregulated miRNAs and seven downregulated miRNAs. Some DE miRNAs were validated via qPCR. A total of 488 putative target genes of the upregulated DE miRNAs were found, and the functional analyses indicated that numerous target genes were enriched in the pathways associated with cancer.

**Discussion:** This suggests that miRNAs of salivary exosomes might have the potential to be used as biomarkers for prediction and diagnosis of lung cancer.

## Introduction

Lung cancer is the leading cause of cancer-related deaths worldwide; two million people are diagnosed with lung cancer, and approximately 1.76 million people die as a result of this disease every year (Hirsch et al., 2017; Thai et al., 2021). Lung cancer is often not diagnosed until it progresses to an advanced stage, and patients have poor treatment outcomes (Nooreldeen and Bach, 2021). Therefore, it is important to explore new ways for diagnosing and treating lung cancer.

Exosomes, secreted by multiple types of cells, are small extracellular vesicles with an average diameter of 30–150 nm, containing RNAs, proteins, and lipids, and play a crucial role in intercellular crosstalk (Kalluri and LeBleu, 2020; Isaac et al., 2021). Previous studies revealed that exosomes were involved in many diseases, including Alzheimer’s disease (Soares Martins et al., 2021), osteoporosis (Shan et al., 2019), and cancer (Yu et al., 2021). In cancer, exosomes are associated with cell proliferation, metastasis, invasion, epithelial–mesenchymal transition (EMT), angiogenesis, and tumor microenvironment (TME) remodeling (Bai et al., 2022). Tumor-derived exosomes contain a variety of stimulatory and inhibitory factors involved in mediating the immune response, which can affect the TME and thus participate in the formation and progression of tumors (Mashouri et al., 2019). Hence, the RNAs, proteins, and lipids from exosomes could be explored as a source of biomarkers for diagnosis and prognosis (Yu et al., 2022). In recent years, significant progress has been made in our understanding of the role of exosomes in the diagnosis, treatment, and prognosis of lung cancer (Li et al., 2021). For example, the deep learning-based spectroscopic analysis of circulating exosomes could be used to diagnose early-stage lung cancer (Shin et al., 2020).

Saliva, an acidic biological fluid composed of secretions from multiple salivary glands, plays a crucial role in numerous biological functions, such as perception of oral sensations, lubrication, chewing, swallowing, and digestion (Kaczor-Urbanowicz et al., 2017). The collection of saliva is convenient for the screening of large populations and provides an alternative for patients whose blood cannot be easily collected or when cooperation is an issue (Chojnowska et al., 2018). In recent years, saliva has been widely used in the diagnosis of many diseases, including Alzheimer’s disease (Ashton et al., 2021), COVID-19 (Fernandes et al., 2020), and cancer (Wang et al., 2017; Li et al., 2022). Saliva-derived exosomal small RNAs have been shown to be useful as biomarkers of esophageal carcinoma (Li et al., 2022), and biomarkers of head and neck cancer were identified through the analysis of cargo and the functional profile of saliva-derived exosomes (Hofmann et al., 2022). In addition, there are studies demonstrating salivary exosomal miRNA-1307-5p is a potential prognosticator for predicting poor survival and poor patient outcome in oral cancers (Patel et al., 2022).

MicroRNAs (miRNAs) derived from exosomes have often been explored as a predictive biomarker for lung cancer, but miRNAs of salivary exosomes remain unexplored. In the current study, we collected saliva from healthy individuals and patients with lung cancer, and salivary exosomes were obtained via a series of centrifugation and ultracentrifugation steps. Subsequently, miRNA sequencing (miRNA-seq), quantitative real-time polymerase chain reaction (qPCR), and bioinformatic analyses were carried out.

## Materials and methods

### Patients and clinical samples

In this study, we aimed to investigate the potential biomarkers for early-stage lung adenocarcinoma by analyzing saliva samples. We collected saliva samples from 18 early-stage (I–IIIA) lung adenocarcinoma patients and 18 non-cancer controls, aged between 50 and 75 years, at Harbin Medical University Cancer Hospital, according to the approved protocols by the Ethics Committee of Harbin Medical University (Supplementary Table S1). In general, having oral diseases will affect the expression of miRNA in saliva (Safari et al., 2023). In order to reduce the interference, the participants who did not have oral diseases were included in the study; meanwhile, age, gender, and smoking and drinking status of the participants in the control group were made as consistent as possible with those in the experimental group. In addition, all subjects provided written informed consent. During the collection period, the participants were seated straight up and instructed to refrain from swallowing or speaking, and approximately 5 mL saliva sample was obtained from each individual.

For each set, six samples were mixed to create one biological replicate and centrifuged at 2,000 g for 5 min at 4°C to discard insoluble materials and cell debris. The samples were stored at −80°C for subsequent use.

### Isolation and identification of exosomes

Saliva samples were centrifuged at 2,500 g for 15 min at 4°C, followed by ultracentrifugation at 120,000 g for 2 h at 4°C to pellet exosomes. Exosome pellets were washed in filtered phosphate-buffered saline (PBS) and re-centrifuged at 120,000 g, the supernatant was removed, and the final pellet was re-suspended in 150 µL of PBS.

Fixation of exosomes was carried out with 2.5% glutaraldehyde for 2 h. Subsequently, the exosomes were purified and suspended in 100 µL serum albumin. Approximately 20 µL of exosomes was overloaded onto a formvar/carbon-coated grid and stained with 3% aqueous phosphor-tungstic acid for 60 s. Exosomes were then examined with transmission electron microscopy (TEM), which indicated their spherical shape.

### Nanoparticle tracking analysis

The supernatants containing particles were analyzed using a NanoSight LM20 instrument equipped with a 640 nm laser (NanoSight, Amesbury, United Kingdom) at 25°C. The movement of particles was tracked using NTA software (version 2.2, NanoSight) with a low refractive index corresponding to cell-derived vesicles. The mean, mode, median particle size, and particle concentration (in millions) for each size were analyzed in each track.

### RNA extraction

Total RNA was extracted from salivary exosomes with TRIzol reagent (Invitrogen; Thermo Fisher Scientific, Inc.) and purified using two phenol–chloroform extractions followed by treatment with RQ1 DNase (Promega, Madison, WI, United States) to remove DNA. The SmartSpec Plus spectrophotometer (Bio-Rad, Hercules, CA, United States) was used to assess the quality and quantity of the purified RNA. The integrity of RNA was further verified by 1.4% agarose gel electrophoresis.

### miRNA-seq

Total RNAs (3 μg) from each sample were used for small RNA cDNA library preparation using a Balancer NGS Library Preparation kit (Gnomegen, San Diego, CA, United States) according to the manufacturer’s protocol prior to directional RNA-seq library preparation. The whole library was subjected to 10% native polyacrylamide gel electrophoresis. Then, the bands corresponding to miRNA insertion were cut and eluted. Ultimately, the purified libraries were quantified using the Qubit Fluorometer (Invitrogen; Thermo Fisher Scientific, Inc.) after ethanol precipitation and washing. The RNA libraries were subjected to 150 bp pair-end sequencing on the HiSeq 2500 platform (Illumina, Inc., San Diego, CA, United States).

The FASTX-Toolkit (version 0.0.13) (http://hannonlab.cshl.edu/fastx_toolkit/) was used to process raw reads for obtaining reliable clean reads. RNAs <16 or >30 nt in length were also removed from the subsequent analysis according to the length of the adapter and mature miRNA lengths. Subsequently, the clean reads were searched against the Rfam database (version 12.0) using Bowtie (Langmead et al., 2009). Matches to transfer RNAs and ribosomal RNAs were discarded. The remaining unique sequences were aligned using Bowtie against the miRBase database (Kozomara and Griffiths-Jones, 2014), with one mismatch allowed. The aligned small RNA sequences were convinced by conserved miRNAs.

To investigate the expression profiles of identified miRNAs, the frequency of miRNA counts was normalized to transcripts per million (TPM) using the following formula: normalized expression = actual read count/total read count × 10^6^. The edgeR (v3.22) package of Bioconductor software (Robinson et al., 2010) was used to explore the DE miRNAs, and fold change (FC) ≥ 2 or ≤0.5 and *p* ≤ 0.05 and false discovery rates (FDRs) ≤ 0.05 indicated a statistically significant DE miRNA. The human miRNA and transcript sequences of miRBase were calculated by applying the miRanda algorithm (Krek et al., 2005). Subsequently, TargetScan was used for predicting putative miRNA targets (Lewis et al., 2005). To explore the gene function and analyze the frequency distribution of functional categories, Gene Ontology (GO) and Kyoto Encyclopedia of Genes and Genomes (KEGG) analyses were employed using the R package “clusterProfiler” using the DAVID bioinformatics database (Huang et al., 2009).

### Western blotting

Salivary exosome proteins were extracted and separated by denaturing sodium dodecyl sulfate-polyacrylamide gel electrophoresis (SDS-PAGE). Subsequently, they were transferred to a polyvinylidene difluoride (PVDF) membrane and incubated with blocking buffer [5% skimmed milk powder in PBS–Tween-20 (PBST)] for 1 h. After washing with PBST, the membrane was incubated with primary antibodies (CD9 and syntenin; Abcam, MA, United States) according to the manufacturer’s instructions. Then, the PVDF membrane was incubated with the appropriate secondary antibody for 1 h followed by washing. Finally, the PVDF membrane was incubated for 3 min with SuperSignal West Pico Western HRP substrate at room temperature and imaged using a Tanon 5200 multi-imaging system (Tanon, Shanghai, China).

### qPCR

qPCR was carried out to detect the expression levels of miRNAs using primers designed for reverse transcription (RT) and qPCR. The primer sequences used in our study were as follows: miR-92b-5p: 5′-CTC AAC TGG TGT CGT GGA GTC GGC AAT TCA GTT GAG CAC TGC AC-3′ (RT), 5′-ACA CTC CAG CTG GGA GGG ACG GGA CGC GGT-3′ (forward), and 5′-TGG TGT CGT GGA GTC G-3′ (reverse); miR-532-3p: 5′-CTC AAC TGG TGT CGT GGA GTC GGC AAT TCA GTT GAG TGC AAG CC-3′ (RT), 5′-ACA CTC CAG CTG GGC CTC CCA CAC CCA AGG-3′ (forward), and 5′-TGG TGT CGT GGA GTC G-3′ (reverse); miR-135b-5p: 5′-CTC AAC TGG TGT CGT GGA GTC GGC AAT TCA GTT GAG TCA CAT AG-3′ (RT), 5′-ACA CTC CAG CTG GGT ATG GCT TTT CAT TCC T-3′ (forward), and 5′-TGG TGT CGT GGA GTC G-3′ (reverse); and U6: 5′-CTC GCT TCG GCA GCA CA-3′ (forward) and 5′-AAC GCT TCA CGA ATT GTG CGT-3′ (reverse). cDNAs were generated by standard procedures, and real-time PCR was performed on Bio-Rad S1000 (Bio-Rad, Hercules, CA, United States) with Bestar SYBR Green RT-PCR Master Mix (DBI Bioscience, Shanghai, China). The PCR cycling conditions were set as follows: 95°C for 10 min, 38 cycles at 95°C for 15 s, annealing, and extension at 60°C for 1 min. qPCR amplifications were performed in triplicate for each sample. Transcript levels for the genes analyzed were measured in comparison with the housekeeping gene U6 as an internal reference standard using the 2^−ΔΔCT^ method (Livak and Schmittgen, 2001).

### Statistical analyses

The data were analyzed for statistical significance using Microsoft Excel (2012). All data are presented as the mean ± standard deviation (mean ± SD). Student’s *t*-test (paired) was used to determine the statistical significance when comparing the means of two datasets. A *p*-value < 0.05 was considered statistically significant.

### Online data acquisition and deposition

In this study, a total of 567 samples, including 521 patients with lung cancer and 46 normal tissues, were obtained from The Cancer Genome Atlas (TCGA) database (https://portal.gdc.cancer.gov/).

The datasets obtained in the current study have been deposited in the National Center for Biotechnology Information under accession number PRJNA908267 (https://www.ncbi.nlm.nih.gov/bioproject/PRJNA908267).

## Results

### Isolation and characterization of salivary exosomes

**FIGURE 1:**
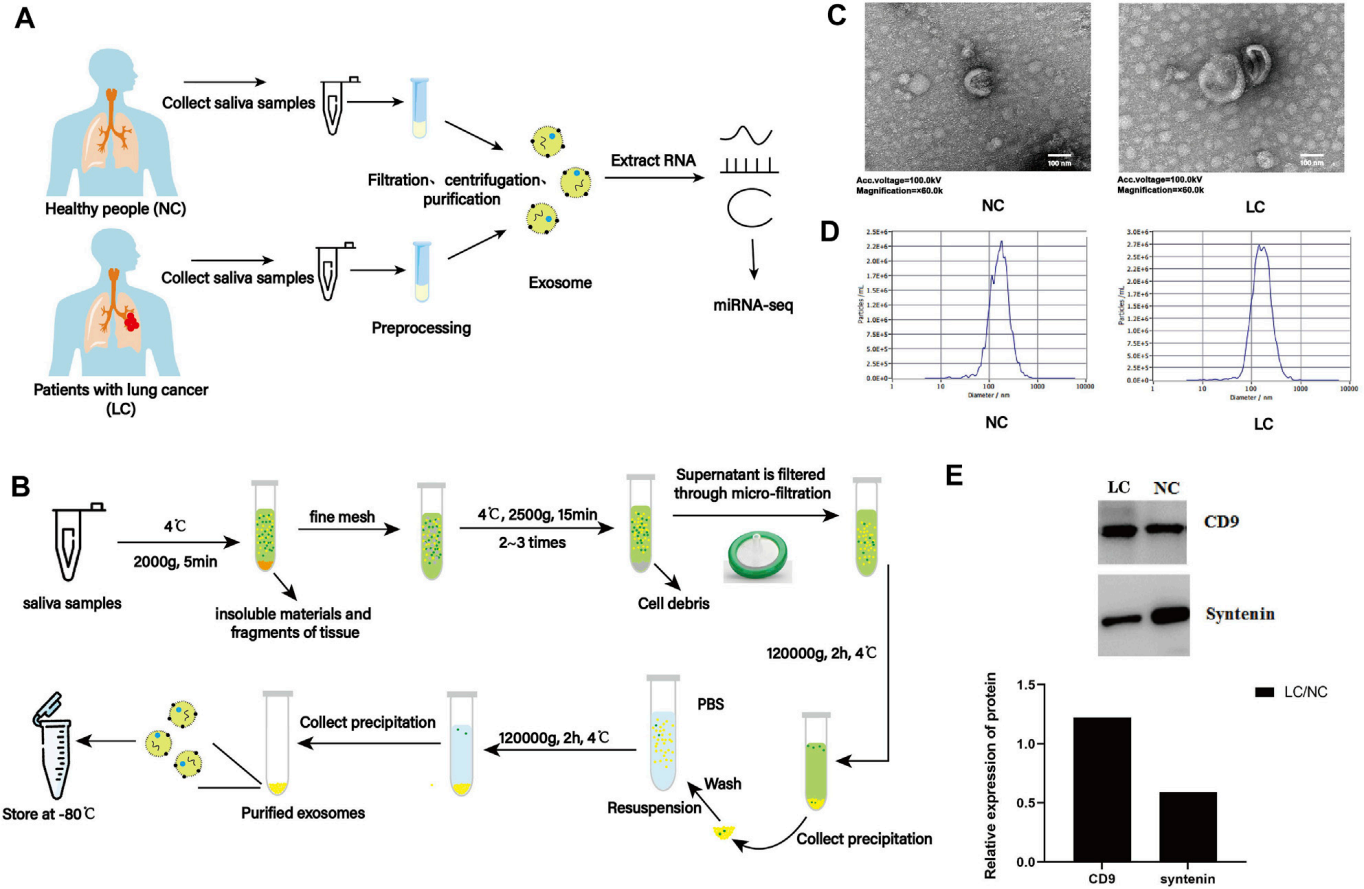
Characterization of salivary exosomes from individuals with (LC) or without lung cancer (NC). **(A)** Schematic diagram illustrating the experimental design from sample collection to sequencing. **(B)** Schematic diagram illustrating isolation of salivary exosomes. **(C)** Exosomes were imaged with transmission electron microscopy (TEM). Electron microscopy allowed visualization of membrane-bound nanovesicles sized ∼100 nm. Scale bar = 100 nm. **(D)** Analysis of exosomes using a NanoSight LM10-HS instrument. **(E)** Exosome validation by Western blotting indicating expression of the exosomal markers CD9 and syntenin.

Saliva samples from 18 patients with lung cancer (LC) and 18 individuals without lung cancer (NC) were collected (Figure 1A). Six samples for each group were mixed to create one biological replicate, and six samples of salivary exosomes (LC-1, LC-2, and LC-3; NC-1, NC-2, and NC-3) were obtained by a series of differential centrifugation, ultracentrifugation, and filtration steps (Figure 1B).

TEM examination indicated that exosomes from two groups showed a characteristic round- or cup-shaped morphology and dimension (Figure 1C). Nanoparticle tracking analysis (NTA) indicated that the average sizes of exosomes from LC and NC groups were 73.34 and 75.68 nm, respectively (Figure 1D). Furthermore, exosomes were validated by Western blotting. Exosomes can be characterized using exosome markers (CD9 and syntenin) expressed on their outer membrane. By analyzing the gray values of marker proteins, it was discovered that the expression level of the CD9 protein within the LC group was higher than that in the NC group. In contrast, the opposite was observed for the expression level of the syntenin protein (Figure 1E). These results indicated that salivary exosomes were isolated successfully and were suitable for further investigation.

### miRNAs of salivary exosomes

Previous studies have indicated that exosomal miRNAs play significant roles in cancer biology and clinical applications (Sun et al., 2018a; Hu et al., 2020). The aim of this study was to investigate the miRNA cargo of salivary exosomes from two groups. Therefore, a total of six small RNA libraries (LC-1, LC-2, and LC-3; NC-1, NC-2, and NC-3) were constructed for miRNA-seq with three biological replicates for each group.

**FIGURE 2:**
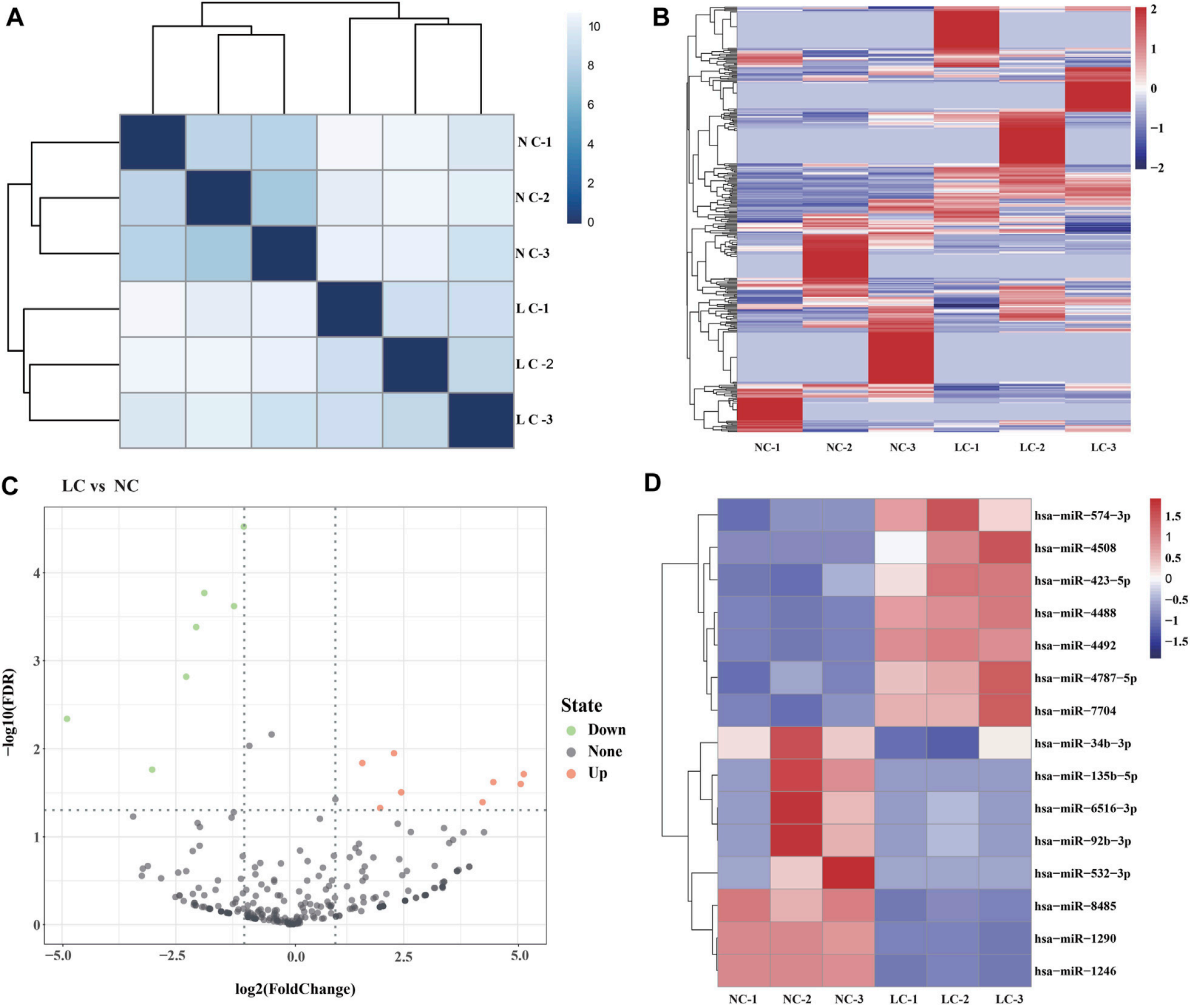
Salivary exosome miRNAs of the salivary exosomes. **(A)** Hierarchical clustering heatmap showing the distinct miRNA expression patterns of the six samples (LC-1, LC-2, and LC-3; NC-1, NC-2, and NC-3). **(B)** Heatmap of the miRNAs identified in the salivary exosomes. **(C)** Volcano plot indicating the expression of miRNAs in LC vs*.* NC groups. X-axis represents the log_2_ fold-changes (FC), while the y-axis represents the −log_10_ significant difference (*p*-value). miRNAs with significant differential expression are shown in red (upregulated) or blue (downregulated). miRNAs that are not significantly differentially expressed are shown in black. A FC of log_2_ ratio ≥2 and *p*-value < 0.05 were set as the threshold to determine differentially expressed miRNAs. **(D)** Heatmap of the DE miRNAs.

Using Illumina HiSeq 2500, >433.1 million reads were generated, corresponding to ∼73.2 million sequence reads per library; the clean reads accounted for ∼87.7%. Hierarchical clustering heatmap analyses indicated that the global expression pattern of miRNAs differed obviously between the LC and NC groups (Figures 2A, B). The clean reads were matched to Rfam and a human reference genome (Ensembl release 102 GRCh38), and the filtered reads were mapped to miRBase. The read counts were normalized to TPM, and a total of 372 miRNAs were identified (Supplementary Table S2).

### Identification of DE miRNAs

DE miRNAs were determined using edgeR. The threshold for screening differentially expressed miRNAs was set as follows: a “fold change (FC) ≥ 2 or ≤ 0.5 and *p* ≤ 0.05 and false discovery rates (FDR) ≤ 0.05.” A total of 15 DE miRNAs were identified in LC vs. NC, including eight upregulated miRNAs and seven downregulated miRNAs, respectively (Figures 2C, D). The highest and lowest FC values were 2^5.13^ and 2^−4.89^, respectively (Supplementary Table S3). These findings indicated that lung cancer is associated with the expression levels of numerous miRNAs in salivary exosomes.

### RT-qPCR validation

**FIGURE 3:**
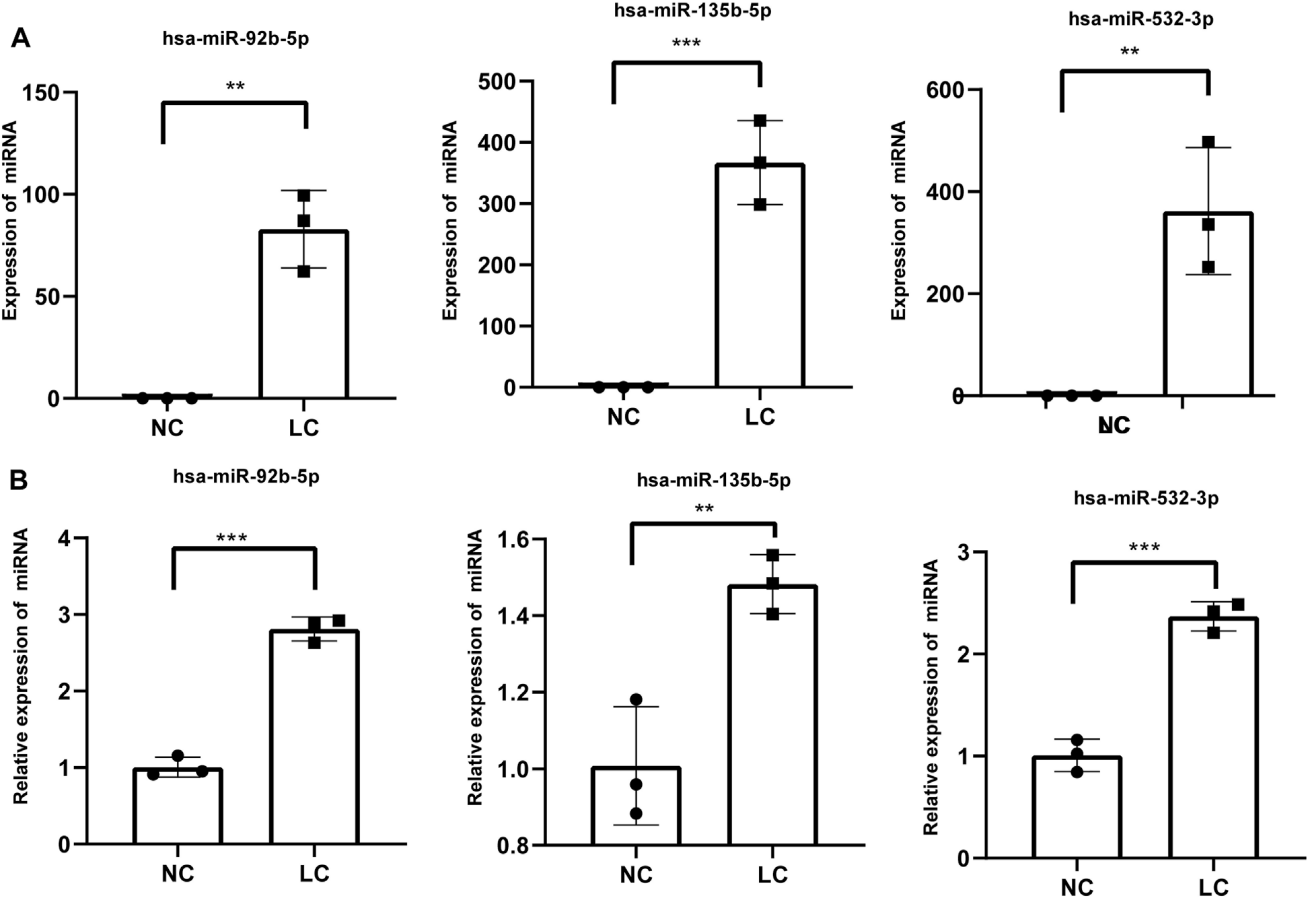
RT-qPCR validation of three differentially expressed miRNAs. **(A)** miRNA expression was determined by high-throughput sequencing. Fragments per kilobase per million mapped reads were used to calculate the expression levels of miRNAs. **(B)** miRNA expression levels of target genes were determined by RT-qPCR and normalized against U6 expression. Statistical analysis was performed by Student’s *t*-test, and data are presented at the mean ± standard deviation of three replicates. *p < 0.05, **p < 0.01. RT-qPCR, reverse transcription quantitative real-time polymerase chain reaction.

Three miRNAs (miR-92b-5p, miR-532-3p, and miR-135b-5p) were selected from these identified miRNAs and verified by RT-qPCR (Figure 3A). The RT-qPCR expression results were highly correlated with the RNA-seq results (Figure 3B). Hence, the RNA-seq data were reliable and suitable for further analysis.

### Prediction of DE miRNA target genes

miRNAs commonly exert their functions by binding to complementary target sites in the mRNAs of their target genes. Using the miRanda algorithm on human miRNA and transcript sequences of miRBase and TargetScan, 488 and 161 overlapped target genes were identified for the upregulated and downregulated DE miRNAs in LC vs. NC (Supplementary Table S4), respectively. This suggests that the DE miRNAs of salivary exosomes have the potential to regulate the expression levels of multiple genes.

### Functional analysis of target genes of upregulated DE miRNAs

**FIGURE 4:**
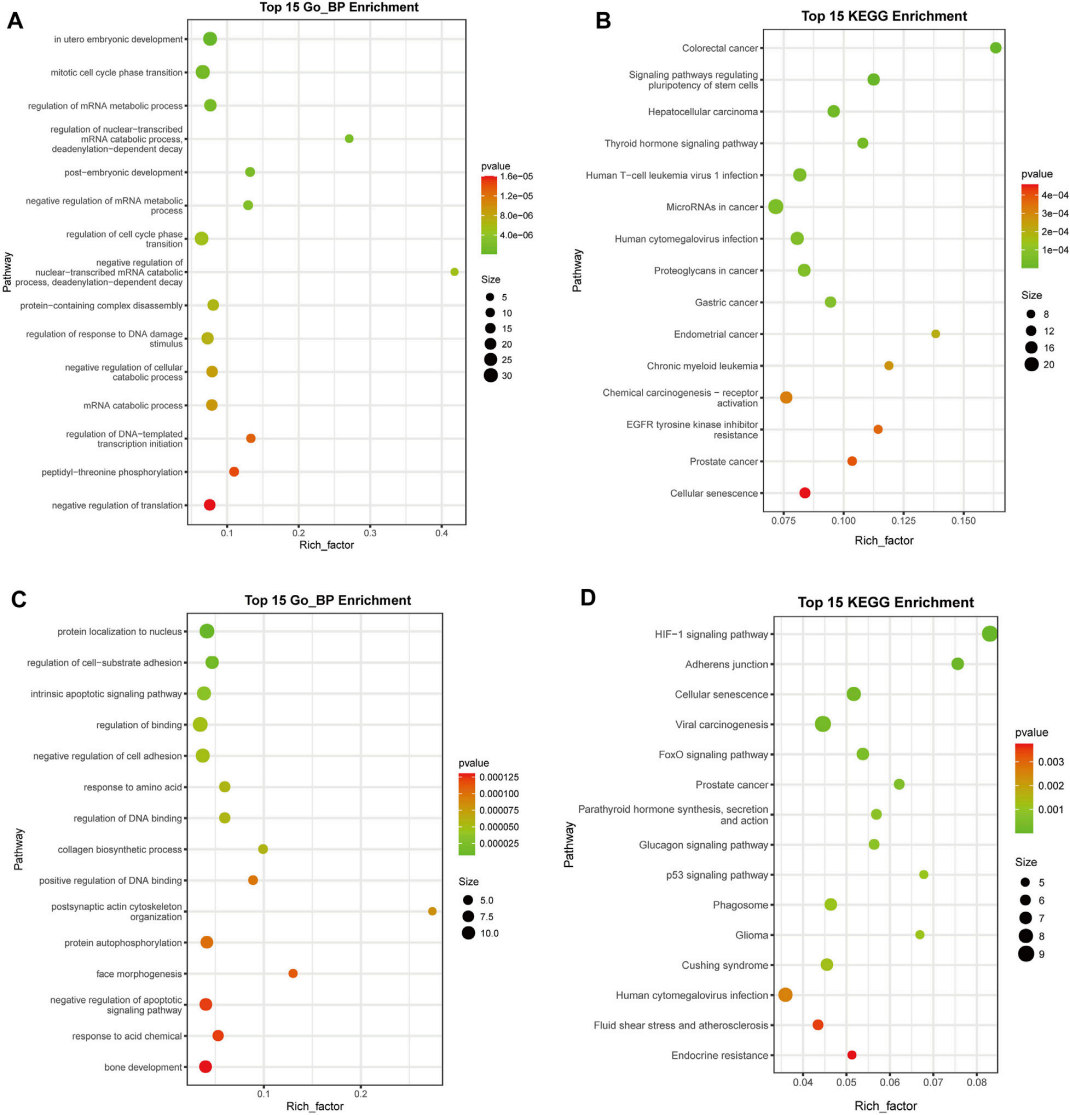
Functional analysis of target genes of the upregulated and downregulated differentially expressed (DE) miRNAs. **(A)** GO analysis of upregulated DE miRNA target genes **(B)** KEGG analysis of upregulated DE miRNA target genes. **(C)** GO analysis of downregulated DE miRNA target genes. **(D)** KEGG analysis of downregulated DE miRNA target genes. The top 15 terms are presented. miRNA, microRNA; GO, Gene Ontology; KEGG, Kyoto Encyclopedia of Genes and Genomes. The rich factor indicates the enrichment degree of terms, while y-axis represents the names of the enriched terms. The area of the node indicates the number of genes. The *p*-value is shown by a color scale with the statistical significance increasing from green to red.

To identify the pathways associated with the target genes of the upregulated DE miRNAs, GO enrichment analysis was conducted and 54 biological pathways were identified (*p* < 0.01, FDR < 0.01) (Figure 4A; Supplementary Table S5). A large number of terms were associated with cell proliferation and cycle, including “mitotic cell cycle phase transition” (GO: 0044772), “regulation of cell cycle phase transition” (GO: 1901987), “regulation of mitotic cell cycle phase transition” (GO: 1901990), “G1/S transition of mitotic cell cycle” (GO: 0000082), “negative regulation of cell cycle” (GO: 0045786), “cell cycle G1/S phase transition” (GO: 0044843), and “negative regulation of mitotic cell cycle” (GO: 0045930). These results suggested that deregulated expression of miRNAs might be associated with a disorder of cell proliferation.

KEGG enrichment analysis revealed that 20 significant functional terms were associated with the target genes of the upregulated DE miRNAs (*p* < 0.01, FDR < 0.01) (Figure 4B; Supplementary Table S6). Multiple enriched pathways were associated with cancer, including “colorectal cancer” (ID: hsa05210), “hepatocellular carcinoma” (ID: hsa05225), “microRNAs in cancer” (ID: hsa05206), “proteoglycans in cancer” (ID: hsa05205), “gastric cancer” (ID: hsa05226), “endometrial cancer” (ID: hsa05213), “chronic myeloid leukemia” (ID: hsa05220), “chemical carcinogenesis-receptor activation” (ID: hsa05207), “prostate cancer” (ID: hsa05215), “breast cancer” (ID: hsa05224), and “bladder cancer” (ID: hsa05219).

Moreover, GO and KEGG enrichment analyses were carried out for downregulated DE miRNAs (Figures 4C, D; Supplementary Tables S7, S8), resulting in 64 and 16 terms, respectively (*p* < 0.01, FDR < 0.05). It was found that multiple enriched GO terms were associated with apoptosis of cell, including “intrinsic apoptotic signaling pathway” (GO: 0097193), “negative regulation of apoptotic signaling pathway” (GO: 2001234), “neuron apoptotic process” (GO: 0051402), “regulation of apoptotic signaling pathway” (GO: 2001233), “intrinsic apoptotic signaling pathway in response to DNA damage by p53 class mediator” (GO: 0042771), and “cellular response to abiotic stimulus” (GO: 0071214), and several KEGG terms were involved with cancer, including “viral carcinogenesis” (ID: hsa05203), “prostate cancer” (ID: hsa05215), and “glioma” (ID: hsa05214).

These results suggested that deregulated expression of miRNAs might be involved in the occurrence and development of cancer.

## Discussion

An improved understanding of cancer is important for the improvement of treatment options (Tarighati et al., 2023). Although tissue biopsy and conventional imaging techniques have been widely used for many years, limitations remain unaddressed. For tissue biopsy, limited sampling is often hard to capture the heterogeneity and evolution of tumors (Cheng et al., 2019), while conventional imaging techniques are less sensitive for the early detection of cancer (Loy et al., 2022). In recent years, liquid biopsy, which aims to provide an alternative to invasive tissue biopsy by analyzing biomarkers in biofluids that reflect the nature of cancer (Ignatiadis et al., 2021), has been widely investigated. In the present study, the collection of saliva samples was convenient as all participants were more cooperative. Therefore, we can use saliva samples as liquid biopsy more widely, and putative biomarkers might be discovered by comparative analyses in future.

Exosomes are small extracellular vesicles secreted by various types of cells that play an essential role in intercellular signaling and cellular homeostasis (Stahl and Raposo, 2019) and affect the pathophysiological conditions of recipient cells. Hence, the molecules derived from exosomes could be used as ideal biomarkers in detecting and monitoring cancer for their biological role in cancer pathogenesis (Mashouri et al., 2019). Exosomes are easily isolated from a wide variety of body fluids, including blood, urine, saliva, breast milk, and cerebrospinal fluid; therefore, exploring exosomal biomarkers of cancer is feasible (Nair et al., 2018; Yu et al., 2022). In the present study, salivary exosomes were isolated by a series of centrifugation and ultracentrifugation steps (Figure 1B), and these protocols could be referenced for future studies.

Exosomes contain diverse constituents including nucleic acids, proteins, lipids, amino acids, and metabolites. miRNAs are non-coding RNAs of approximately 21–23 nucleotides in length that can regulate gene expression at the posttranscriptional level. Exosomal miRNAs, which are transferred into extracellular vesicles through unknown mechanisms, are not prone to RNase-mediated degradation (Yu et al., 2016; Garcia-Martin et al., 2022). Hence, miRNAs are more stable and have been intensely investigated compared with other molecules derived from exosomes (Sun et al., 2018b; Lin et al., 2022). In this study, using miRNA-seq and comparative analyses, a total of 372 exosomal miRNAs were identified, and it was also found that eight miRNAs were significantly upregulated in the salivary samples of patients with lung cancer (Figures 2, 3), suggesting exosomal miRNAs might be suitable biomarkers for disease diagnosis.

Functional analyses of these putative targets of the upregulated miRNAs suggested that these miRNAs might regulate pathways associated with cancer (Figure 4). In the present study, miRNA-Seq and RT-qPCR analyses indicated that miR-92b-5p was more highly enriched in salivary exosomes from individuals with lung cancer (Figure 3). Previous studies suggested that miR-92b-5p could be used for predicting the prognosis of gastric cancer (Xu et al., 2021) and biliary tract cancer (Høgdall et al., 2022), suggesting that this exosomal miRNA might have the potential to be used as a diagnostic biomarker of lung cancer. Although other studies have also associated miR-532-3p with cancer (Han et al., 2019; Jiang et al., 2019), salivary exosomal miR-532-3p deserves further investigation.

Moreover, we obtained 567 samples, including 521 patients with lung cancer and 46 normal tissues, from the TCGA-LUAD database (Supplementary Table S9). The expression levels of some miRNAs were analyzed, and the results indicated that three miRNAs (miR-92b-5p, mir-135b-5p, and mir-532-3p) were upregulated in tumor samples (Supplementary Figures S1A–C). Subsequently, logistic regression analysis was used to predict the effect of the model constructed by these three indicators (hsa-mir-92b-5p, hsa-mir-532-3p, and hsa-mir-135-5p) in distinguishing patients with lung adenocarcinoma from normal people; four models [mir-92b-5p (model 1), has-mir-532-3p (model 2), hsa-mir-135b-5p (model 3), and mir-92b-5p + hsa-mir-532-3p + mir-135b-5p (model 4)] were established and analyzed, and the results indicated that AUCs (areas under the curve) of four models were 0.590 (*p* < 0.001), 0.699 (*p* < 0.001), 0.906 (*p* < 0.001) and 0.912 (*p* < 0.001), respectively (Supplementary Figure S1D). At the same time, it can be seen that the combined use of multiple miRNAs can achieve better results than a single miRNA, suggesting that they might be useful as biomarkers for lung cancer prediction and diagnosis.

## Data Availability

The datasets presented in this study can be found in online repositories. The names of the repository/repositories and accession number(s) can be found in the article/Supplementary Material.
