# Supplementary material for: Comparative analyses of salivary exosomal miRNAs for patients with or without lung cancer
Source: Front Genet. 2023 Nov 3;14:1249678. doi: 10.3389/fgene.2023.1249678 (PMC10657645; doi:10.3389/fgene.2023.1249678)
Supplement: Supplementary file 1 [file DataSheet1.zip › Supplementray data/Figure S1.docx]

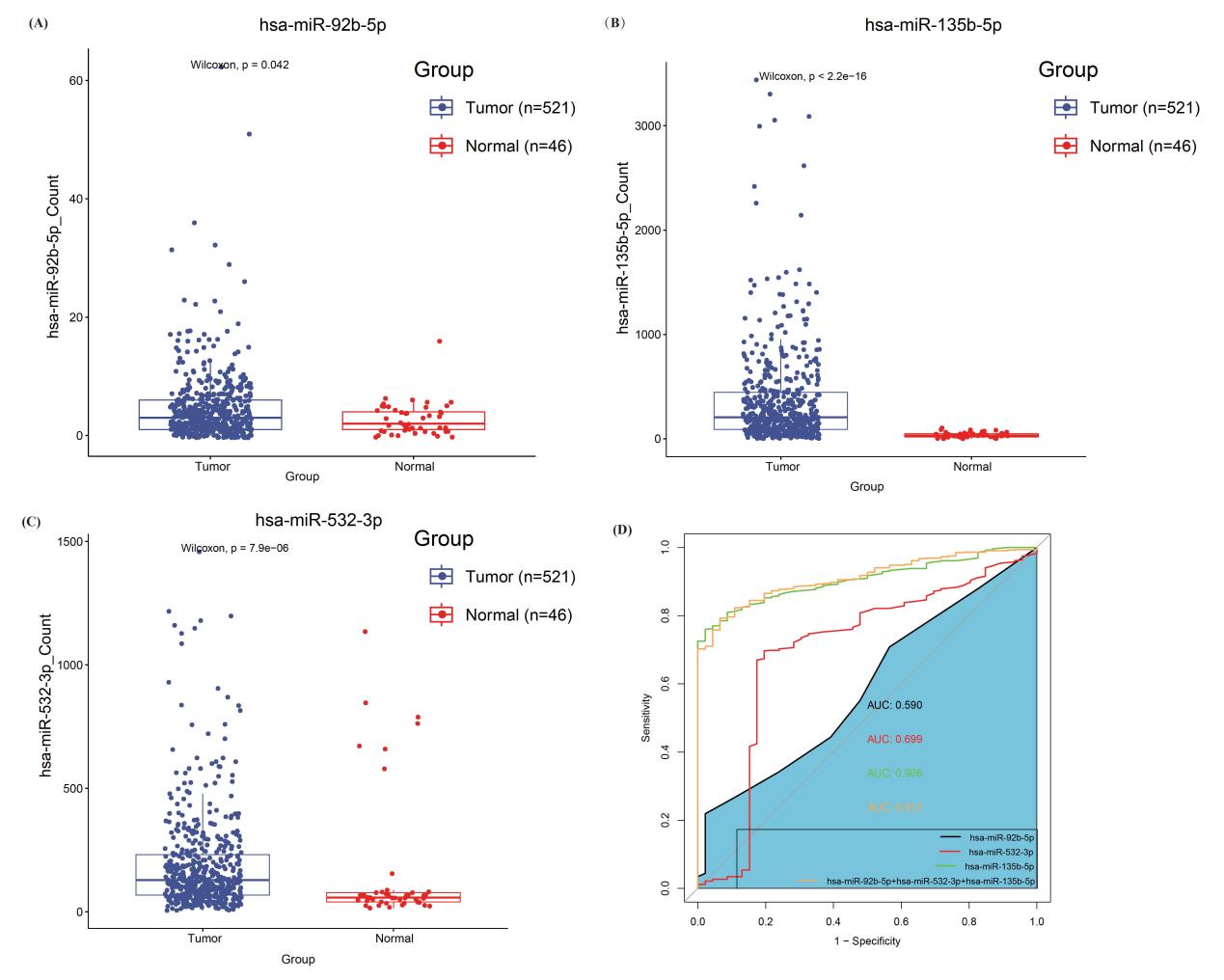


Figure S1. Analysis the relationships between the expression levels of miRNAs and clinical features using the LUAD data set in TCGA database. (A), (B) and (C). The comparative analyses of the expression levels of miR-92b-5p, miR-135b-5p and miR-532-3p, respectively. (D) Evaluation of the models [miR-92b-5p (model 1), miR-532-3p (model 2), miR-135b-5p (model 3), miR-92b-5p + miR-532-3p + miR-135b-5p (model 4)] based on ROC (receiver operating characteristic curve) curve.
