# Supplementary figures and images for: Comparative analyses of salivary exosomal miRNAs for patients with or without lung cancer
Source: Front Genet. 2023 Nov 3;14:1249678. doi: 10.3389/fgene.2023.1249678 (PMC10657645; doi:10.3389/fgene.2023.1249678)

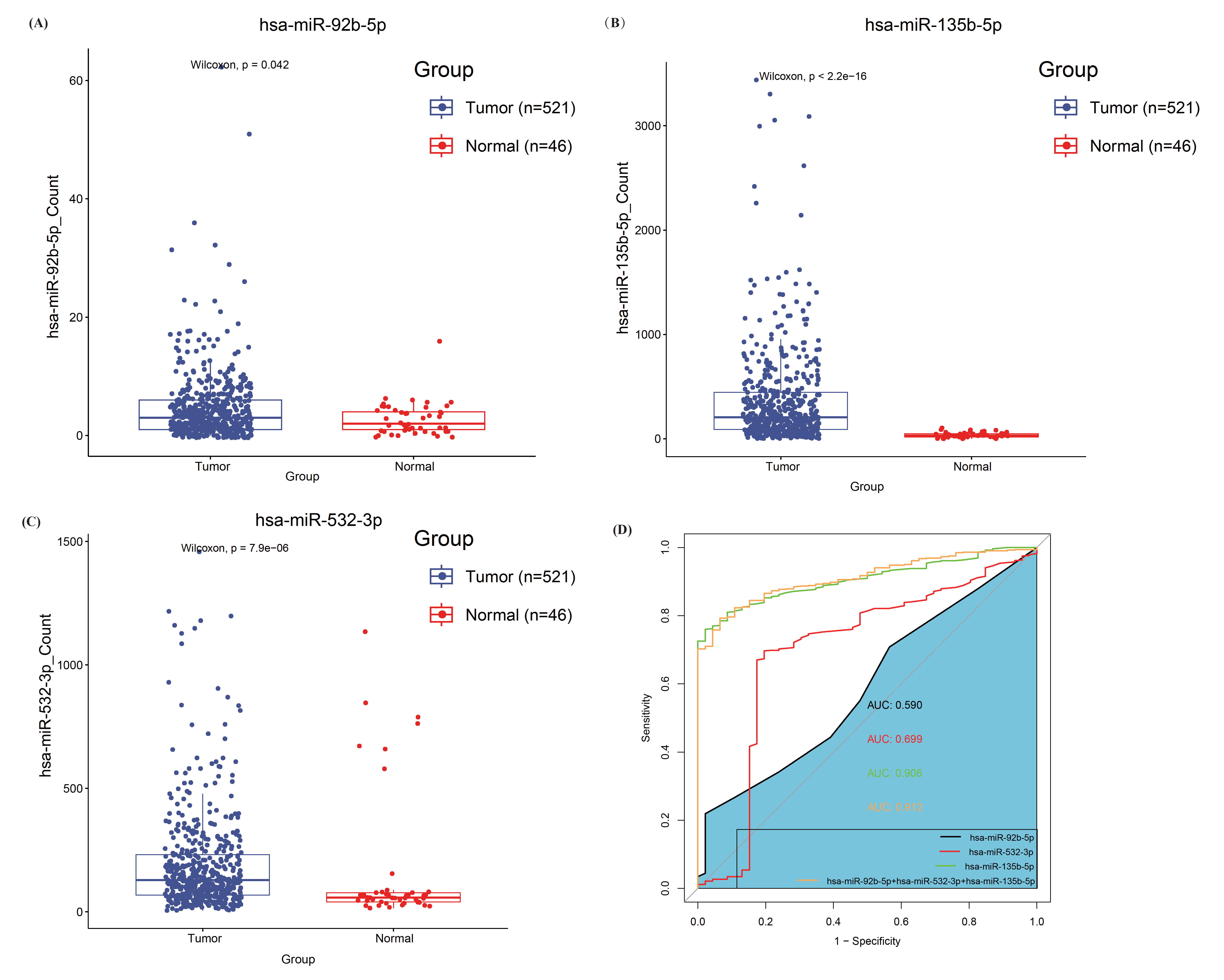

Supplement: Supplementary file 1 [file DataSheet1.zip › Supplementray data/Figure S1.jpg]
